# Low infra red laser light irradiation on cultured neural cells: effects on mitochondria and cell viability after oxidative stress

**DOI:** 10.1186/1472-6882-9-8

**Published:** 2009-04-15

**Authors:** Alessandro Giuliani, Luca Lorenzini, Michele Gallamini, Alessandro Massella, Luciana Giardino, Laura Calzà

**Affiliations:** 1 BioPharmaNet-DIMORFIPA, University of Bologna, Via Tolara di Sopra 50, 40064 Ozzano dell'Emilia, Bologna, Italy; 2 RGMD-Medical Devices Division, Via Rolla 13/13 16152 Genova, Italy; 3 National Institute of Biostructures and Biosystems (INBB), Viale medaglie d'Oro 305 – 00136 Roma, Italy

## Abstract

**Background:**

Considerable interest has been aroused in recent years by the well-known notion that biological systems are sensitive to visible light. With clinical applications of visible radiation in the far-red to near-infrared region of the spectrum in mind, we explored the effect of coherent red light irradiation with extremely low energy transfer on a neural cell line derived from rat pheochromocytoma. We focused on the effect of pulsed light laser irradiation vis-à-vis two distinct biological effects: neurite elongation under NGF stimulus on laminin-collagen substrate and cell viability during oxidative stress.

**Methods:**

We used a 670 nm laser, with extremely low peak power output (3 mW/cm^2^) and at an extremely low dose (0.45 mJ/cm^2^). Neurite elongation was measured over three days in culture. The effect of coherent red light irradiation on cell reaction to oxidative stress was evaluated through live-recording of mitochondria membrane potential (MMP) using JC1 vital dye and laser-confocal microscopy, in the absence (photo bleaching) and in the presence (oxidative stress) of H_2_O_2_, and by means of the MTT cell viability assay.

**Results:**

We found that laser irradiation stimulates NGF-induced neurite elongation on a laminin-collagen coated substrate and protects PC12 cells against oxidative stress.

**Conclusion:**

These data suggest that red light radiation protects the viability of cell culture in case of oxidative stress, as indicated by MMP measurement and MTT assay. It also stimulates neurite outgrowth, and this effect could also have positive implications for axonal protection.

## Background

Considerable interest has been aroused in recent years by the well-known notion that biological systems are sensitive to visible light. This interest has generated research and technical development in different directions, including basic science and medical applications. A strong impulse to this old idea was given by the introduction of lasers as a light source, which offers many benefits, as a laboratory and clinical tool, such as mono-chromaticity and the possibility of transport by means of fibres. In fact, therapeutic applications of low level lasers in many medical conditions involving not only skin [1,2] have expanded considerably over the last ten years, increasing the demand for a better understanding of its cellular and molecular effects.

LLLT (low level laser therapy, including phototherapy and photostimulation) has been shown to modulate biological processes, depending on the power density, wavelength, and frequency, and to have positive effects on wound healing, on improving angiogenesis, on muscle regeneration and diabetic wounds repair [3,4] Moreover, the histological analysis of tissue indicates that laser irradiation shortens the inflammatory phase as well as accelerating the proliferative and maturation phase, and positively stimulates the regeneration of injured epidermis and the reparation of injured striated muscle [5]. The pioneering work of Tiina Karu [6-8] has defined critical parameters in this rapidly growing area governing wavelengths, output power, continuous wave or pulsed operation modes, pulse parameters, coherence and polarization, and has also indicated possible biological light acceptors at organic, cellular, subcellular and molecular level On the basis of these extensive studies it has been proposed that the terminal enzyme of the respiratory chain cytochrome c oxidase located in mitochondria acts as photoacceptor for the red-to-near IR region in eukaryotic cells, and the modulation of the redox state of the mitochondria generates secondary reactions through cell signalling molecules [9].

Also in view of the clinical application of visible radiation in the far-red to near-infrared region of the spectrum [10] there is an increasing interest in studying the effects of visible radiation on simplified biological systems, such as cultured excitable cells. In this paper we explored the reaction of a well established neural cell line (PC12) to coherent red light irradiation (670 nm) with extremely low energy transfer (20 mW/cm^2^). We focused on the effect of pulsed light laser irradiation in two distinct biological effects: neurite elongation under NGF stimulus on a laminin-collagen substrate and mitochondria membrane potential and activity under basal conditions and after oxidative stress. The latter experiment was performed in living cells using the live dye JC1 and single fluorescence laser microscopy [11].

## Methods

### PC 12 cell culture

Rat pheochromocytoma cell line 12 (PC12) (clone BSTCL91, Istituto Zooprofilattico Sperimentale della Lombardia e dell'Emilia, Brescia, Italy) was cultured in DMEM (GIBCO) supplemented with 10% horse serum (GIBCO), 5% FBS (GIBCO), 2 mM glutamine, 100 units/ml penicillin, and 100 μg/ml streptomycin at 37°C in a 5% CO2, incubator. In order to study neuritis elongation, cells were seeded at 5 × 10*3 cells/well on 24 multi-well plates and differentiated by treating with NGF (10 ng/ml; a generous gift from Dr. L. Aloe, Inst. Neurobiol. Mol. Med., CNR, Rome, Italy; [12] in DMEM supplemented with 0.5% FBS 1% horse serum. Medium was changed every 3 days.

For MTT assay cells were seeded at 5 × 10*4 cell/well on 4 multi-well plates coated with Poly-L-Lysine (10 microg/ml; SIGMA). Oxidative stress and laser treatment were performed 24 h after seeding. For JC-1 assay cells were seeded at 5 × 10*4 cells/well on a chambered cover glass (Nunc Lab-Tek Chambered Cover glass, Nunc International, NY, USA) coated with Poli-L-Lisyne. Oxidative stress and laser treatment were performed 24 h after seeding. Coverslips or culture wells were first coated with collagen (Collagen Type IV; 0.1 mg/ml; SIGMA) and then recoated with laminin (100 microg/ml; SIGMA).

### Exposure system

**Figure 1:**
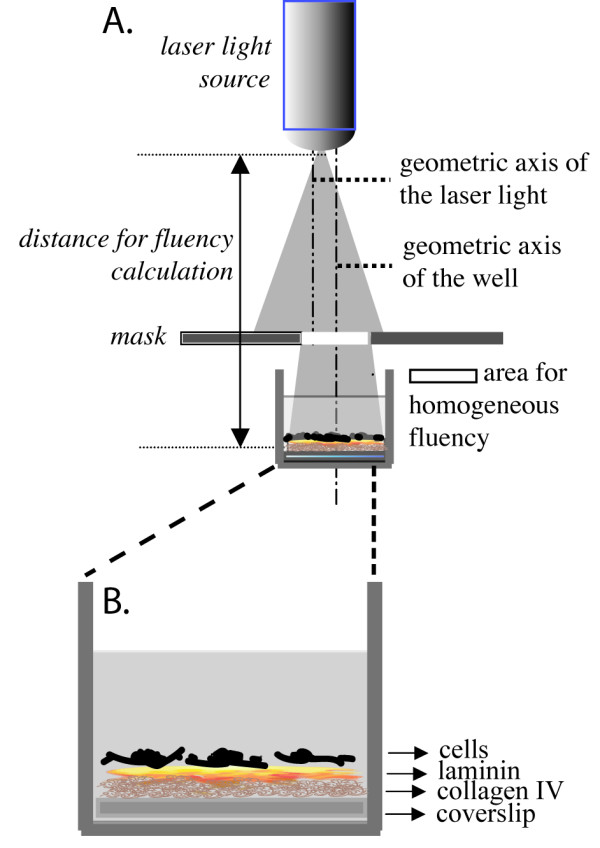
**Representation of laser irradiation system**. A. Figure illustrating the experimental set-up with the main technical features of the beam used for red laser in vitro irradiation. Since the portion of the laser beam with homogeneous power is not coincident with the geometric axis of the laser beam, a black mask interposed between laser source and cells was used to obtain the homogeneous fluency of the beam. B. The cell culture substrate, composed of glass coverslip coated with collagen and laminin, is also shown.

A SANYO DL3149-055A diode laser (on a probe designed and built by RGM, Genoa Italy) was used for irradiation (Fig. 1A). The technical characteristics of this light source were as follows: wavelength (λ) = 670 ± 10 nm; power = 3 mW (peak). In the RGM probe a converging/diverging lens provides a conical beam. As the power density is not homogeneous within the diode laser beam (see Fig. 1B) the distance was adjusted in such a way that over a round spot of 2 cm2 (area of the cell culture well) the light intensity could be considered as visually homogeneous. It has been estimated that only some 75% of the emitted power was thus applied.

The following emission modes were used: MOD 1 = Square Wave Pulsed at 100 Hz Duty Cycle (DC) 1%; MOD 2 = Double Square Wave Pulsed 100 Hz DC 1% + 1 Hz DC 50%. The exposure time, controlled by a microprocessor, was 20 sec or 15 min. Mean Power, Power Density or Fluence, Total Energy and Energy Density in the different emission modes and exposure times are reported in Table 1

**Table 1:** Physical characteristics of laser emission modes

	power		energy		energy	
			
	mean (mW)	density (mW/cm2)	total (mJ)	density(mJ/cm2)	total (mJ)	density (mJ/cm2)
		
Irrad. time	20 sec	20 sec	20 sec	20 sec	15 mi	15mi
		
MOD1	0.02250	0.011250	0.450	0.2250	20.250	10.125
		
MOD2	0.011250	0.005625	0.2250	0.1125	10.125	5.062

Mean power, power density or fluence, total energy and energy density in the different emission modes used in the experiments.

**Figure 2:**
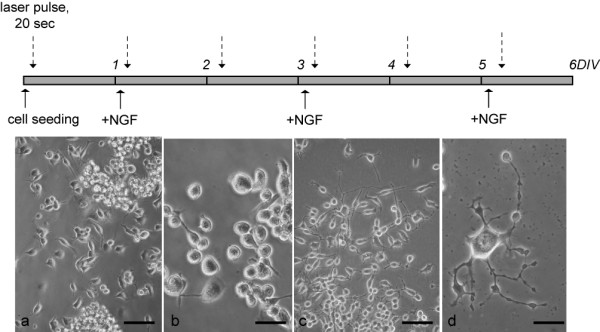
**Schedules for neurite elongation experiment**. The schedules for neurite elongation experiments including culture conditions and laser light irradiation are shown in upper part of the figure (see text for further details). The dotted arrows indicate the daily exposure to 20 sec laser pulse. Micrographs show PC12 cells cultured without (a, b) and with (c, d) NGF in the medium. Scale bar: a, c 100 μμ b, d 50 μμ. Pictures of living cells were taken every days, after laser irradiation. *Abbreviations: DIV, days in vitro; NGF, nerve growth factor*.

The schemes used for laser irradiation in the different experiments are reported in Fig. 2.

### Oxidative stress

In order to challenge cells with an oxidative stress, 10 μl H_2_O_2 _(final concentration 300 μM) was added to each well immediately before laser treatment. Cells were then exposed to laser irradiation for 20 sec or 15 min.

### Proliferation and viability assay

MTT assay is a biochemical cell viability test based on the ability of the mitochondria to reduce the tetraziolium salt 3-(4,5-dimethylthiazol-2-yl)-2,5-diphenyl tetrazolium bromide (MTT, SIGMA) to formazan [13]. Fifteen min after H_2_O_2 _supplementation, growth medium was replaced with 500 μl of OPTI-MEM serum medium without phenol red (GIBCO) and MTT-stock solution (diluted in PBS) was added to each well to give a final concentration of 0.5 mg/ml. After 3 h of incubation at 37°C, the formazan crystals formed were dissolved with 500 μl 10% Triton X-100 in 0.1 N HCl/isopropanol. The absorbance value was measured at 570 nm (Microplate Reader 680, BIORAD, Hercules, CA).

### Neurites elongation analysis

Cells were seeded as described above; serum freshly made with NGF was changed every 3 days and a single laser pulse was performed every day. Every days, after laser irradiation, pictures of living cells were taken with an inverted Olympus IX70 microscope. For each time and laser irradiation schema, 2 different wells were analyzed. For each well 5 frames were captured (20× objective) for a total of 150 cells for each time point. Cells with neurites were defined as those bearing a process twice as long as the cell body length. Neurite length was measured using Image Pro Plus software (Media Cybernetics, MD, USA).

### JC-1 staining of mitochondria membrane potential assay

Mitochondrial Membrane Potential was detected using the MitoPT™ Kit (Mitochondrial Permeability Transition Detection Kit, Immunochemistry Technologies, LLC) incorporating the JC-1 cationic dye. JC-1 was reconstituted in DMSO (100× stock solution), stored at -20°C and used for experiments as 1× solution in serum free medium. Mitochondrial permeability transition events were recorded using time lapse software in a confocal laser scanning microscope (CLSM) (Olympus Fluoview 500; Olympus Optical Co (Europa) GMBH) mounted on an inverted microscope (Olympus IX81) equipped with Ar (λ = 488 nm), Green-HeNe (λ = 543 nm) laser and incubator (Evotec Technologies/PerkinElmer Waltham, MA) (37°C, CO_2_5%, 60% humidity). Cells seeded on a chambered cover glass for 24 h were incubated 15 min with Mito-PT solution (1× Mito-PT in serum free medium) at 37°C in a CO_2_ incubator and then washed once with DMEM. Cells in serum-free medium were exposed to oxidative stress and laser irradiation (see above). For time-lapse analysis of vital mitochondria staining with JC-1 cells were excited with Ar laser (488 nm) and observed with a 560 nm filter. The focus plane was set up to include both nucleus and cytoplasm. Acquisition started immediately after H_2_O_2 _addition, at the same time as laser exposure, and images were taken at 120 sec time intervals for 15 min with a PLAN APO 60X/1.35/oil objective and ×2 zoom (image size 800 × 600). Red laser exposure lasted for 20 sec or 15 min. Each cell included in the frame limit was processed with the FluoView Time Course software, and 15–20 cells were analyzed in each experimental session. Briefly, the mean intensity on a scale ranging from 0 (black) to 4095 (white) was measured each time using the fast XY acquisition mode (scan speed: 1.08 s/scan). Measurements for photo bleaching were also performed in the same experimental session. The time-dependent variation of fluorescence intensity from 120 to 960 sec was then calculated for each cell in the absence (photobleaching) and in the presence (oxidative stress) of H_2_O_2 _and these single cell values were used for statistical analysis.

Maximum photomultiplier voltage was applied to decrease the required laser power as much as possible. The confocal aperture (C.A.) for Ar laser was 105 μm. The Ar laser was used at 40–50% of maximum power, resulting in 4–5 mV energy transfer/observation.

### Statistical analysis

Descriptive statistics are expressed as mean + SEM. One-way ANOVA and post-hoc Tukey's Multiple Comparison Test, and Student's t test were used to compare experimental groups. Results were considered significant when the probability of their occurrence due to chance alone was less than 5%.

## Results

### Neurite elongation

**Figure 3:**
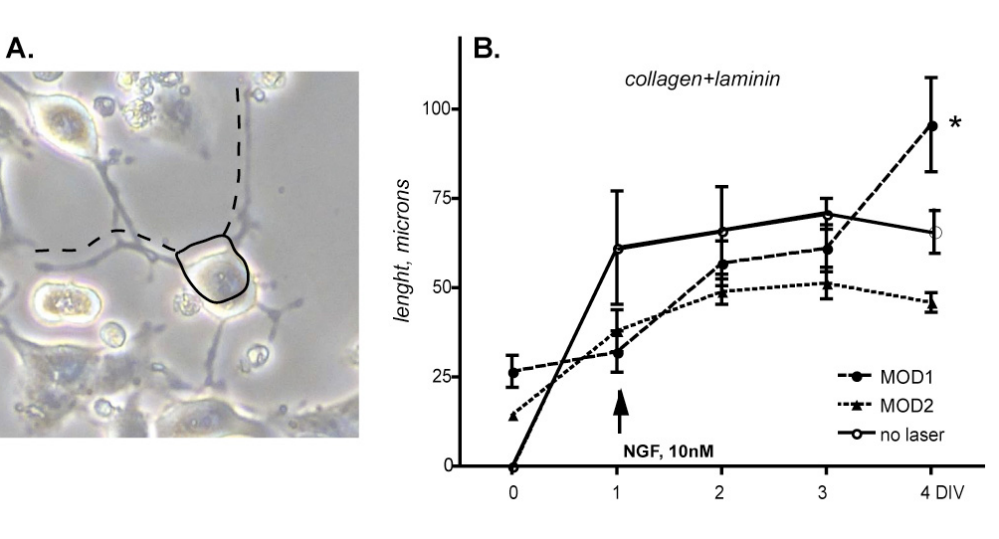
**Effect of laser light irradiation on neurite elongation**. The figure illustrates the effect of laser light irradiation on neurite elongation in PC12 cells cultured on laminin and collagen. A. The continuous black line delineates the cell body, whereas the dotted lines mark the processes (see text for details and inclusion/exclusion criteria). B. Neurite length of non-irradiated, MOD1 and MOD2 irradiated cells at 1, 2, 3 and 4 days in vitro. NGF stimulates neurite outgrowth for 3–4 days; MOD1, but not MOD2, further extends neurite outgrowth. Statistical analysis: two-way ANOVA and post-hoc test, *p < 0.05.

PC12 cells, when they adhere to the substrate, begin differentiation by emitting branched neurites (Fig 3A). As soon as cells are plated (0 DIV), few cells show neurites. As soon as they adhere to the collagen/laminin-coater plates, neuritis growth (1 DIV). At this time, no significant differences were observed between exposed and not exposed cells. Neurite growth is strongly facilitated by the presence of collagen and laminin as substrate coating agents and by the presence of NGF in the medium. In fact, in these culture conditions, neurite growth continues for 3 days. Red light laser irradiation applied according to MOD1 (Square Wave Pulsed at 100 Hz Duty Cycle (DC) 1%) further stimulates neurite outgrowth (two-way ANOVA and post-hoc test *p < 0.05) (Fig. 3B).

### Oxidative stress

In order to evaluate the effect of coherent red light irradiation on cell reaction to oxidative stress, we used two well validated tests, one measuring the mitochondria membrane potential in live cells (by JC-1 fluorescence dye), and one measuring cell viability through a mitochondria-dependent assay (by MTT biochemical test).

**Figure 4:**
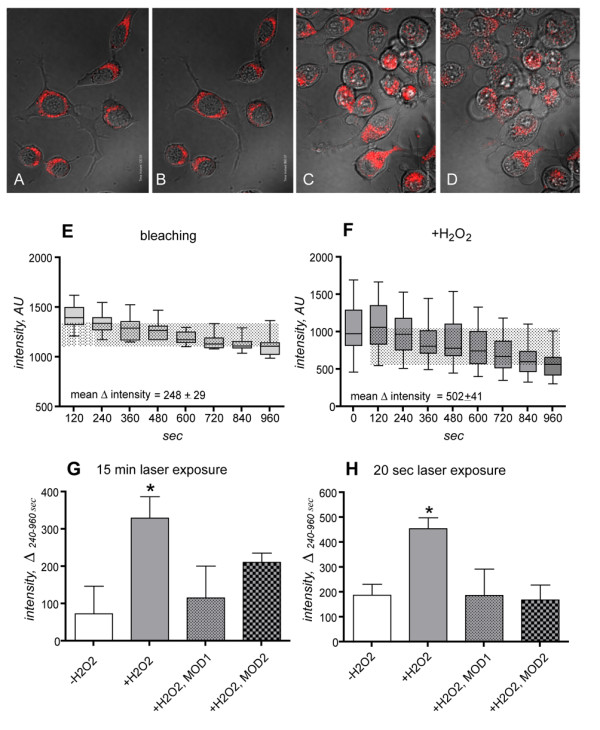
**Effect of laser light irradiation on mitochondria membrane potential**. The figure shows results from mitochondria function experiments. Micrographs A-D illustrate JC-1 accumulation in mitochondria of healthy cells (A, B) and of cells exposed to H_2_O_2 _(C, D), at the beginning (A, C, t = 120 sec) and end (B, D, t = 960 sec) of the observational period. E, F: graphs represent data from a typical experiment, showing the decrease over the time of fluorescence intensity values over the observational time in the absence (photobleaching due to confocal laser stimulation) and at the presence of H_2_O_2 _(oxidative stress). G, H: Δ intensity illustrates the effect of a single 20 sec (H) and 15 min (G) laser light irradiation on mitochondria membrane potential during H_2_O_2_-induced oxidative stress. Both MOD1 and MOD2 exposure to red light radiation prevent MMP variation due to oxidative stress. Statistical analysis: one-way ANOVA and post-hoc test, *p < 0.05.

JC-1 (5,5',6,6'-tetrachloro-1,1',3,3'-tetraethylbenzimidazolo carbocyanine iodide) [14,15] is a lypophilic, cationic dye that can selectively enter mitochondria and reversibly change colour from green to red as the membrane potential increases. In healthy cells with high mitochondrial Δψμ, JC-1 spontaneously forms complexes known as J-aggregates with intense red fluorescence. When the mitochondrial ΔΨ collapses in apoptotic cells the JC-1 aggregates gradually exit the mitochondria and are distributed throughout the cell, assuming a monomeric form, which fluoresces in green. The intensity of the red fluorescent signal was used to evaluate mitochondria vitality. Under the observation conditions used in our experimental set-up, red fluorescence intensity decreases as soon as the mitochondria swell and degenerates after addition of H_2_O_2 _to the medium. Micrographs in Fig. 4 show healthy (A, B) and H_2_O_2 _exposed (C, D) specimens at the beginning (120 sec, A, C) and end (960 sec B, D) of the observational time. Results from a typical experiment reporting fluorescence intensity values over the observational time in the absence of H_2_O_2 _(photobleaching due to confocal laser stimulation) and in the presence of H_2_O_2 _(oxidative stress) are reported in Figs. 4E and 4F, respectively. The mean ΔΨ (difference in mitochondria membrane potential, MMP) in the absence of H_2_O_2 _is 248 ± 29 (corresponding to 20% of the maximal fluorescence), and in the presence of H_2_O_2 _is 502+41 (corresponding to 40% of the maximal fluorescence), thus confirming ongoing oxidative stress in the cells (one-way ANOVA and post-hoc test, *p < 0.05). The difference between the intensity values at the beginning (120 sec) and end (960 sec) generate the ΔΨ values reported in graphs G and H, where the effect of a single 20 sec laser light irradiation, and of 15 min laser light irradiation, respectively, are reported. Both MOD1 and MOD2 irradiation decreases ΔΨ after H_2_O_2 _to control (-H_2_O_2_) values, thus suggesting a protective effect of red light radiation on early mitochondria potential variation due to oxidative stress.

**Figure 5:**
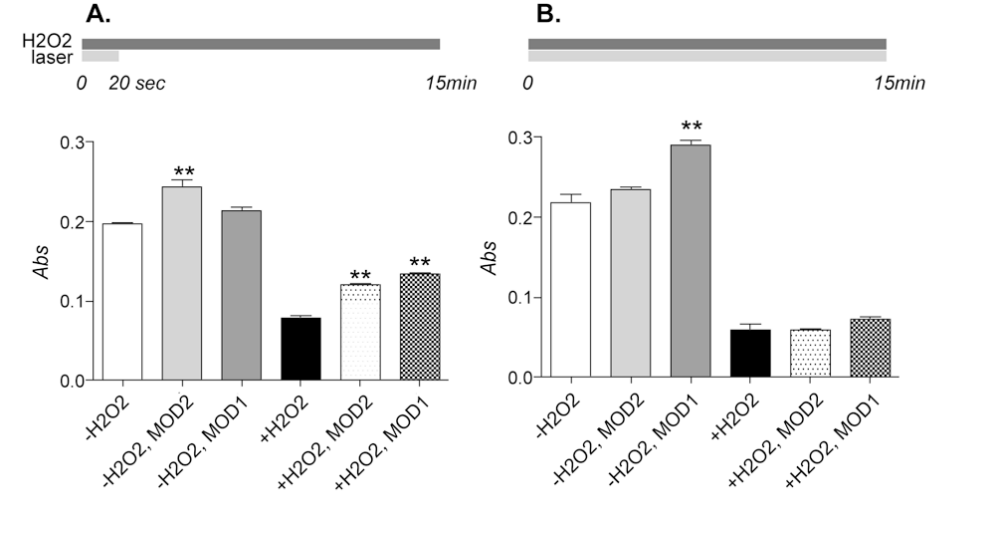
**Effect of laser light irradiation on cell survival**. Overall effect of MOD1 and MOD2 laser light irradiation on cell survival as evaluated by MTT test are illustrated in the figure, where A refers to short (20 sec) and B refers to long (15 min) irradiation. Short laser irradiation using both MOD1 and MOD2 slightly but significantly protects cell culture viability, whereas long laser exposure has no protective effect. Statistical analysis: one-way ANOVA and post-hoc test, *p < 0.05.

We then explored overall cell viability after H_2_O_2 _in PC12 cells exposed to laser irradiation by means of MTT assay (Fig. 5). We found a slight, not significant effect of laser exposure to cell viability in basal conditions. H_2_O_2 _induces a severe decrease in cell viability and red light 20 sec laser irradiation at the beginning of H_2_O_2 _exposure using both MOD1 and MOD2 slightly but significantly protects cell culture viability.

## Discussion

In these experiments we exposed neural cells to a 670 nm laser, with extremely low peak power output (3 mW) and at an extremely low dose (0.45 mJ), 75% of which reached the cells in the culture. This λ corresponds to one of the four suggested "active zones" (peak positions between 667.5 and 683.7 nm) for the investigation of cellular mechanisms of phototherapy [16]. The total energy was approximately 2000 times less than in photodynamic therapy. We also compared different irradiation times (20 sec and 15 min) applied to a well established neuronal cell culture, e.g. PC12.

### Neurite outgrowth

PC12 cells express NGF receptors and, under NGF stimulation, the proliferation rate decreases and neural differentiation takes place [17]. Cell growth is regulated by the adhesive interaction of cell surface and the substrate, which is required for in vitro differentiation. A large number of molecules belonging to the membrane and matrix domains are involved in cell-matrix adhesion and de-adhesion and the dynamic regulation of this interaction regulates key processes, such as cell growth and differentiation [18]. Neurite outgrowth is also dependent on cell adhesion [19], the matrix protein laminin promotes neurite outgrowth [20] and also NGF-mediated neurite outgrowth and elongation are potentiated in cells plated on collagen and laminin coated surfaces [19,21]. In our experiments, PC12 cells were differentiated by NGF on collagen and laminin coated wells. Laminin has a strong, dose-dependent effect on both neurite length and outgrowth and a substrate coating made by laminin and collagen 1 increases the overall volume outgrowth (reflecting neurite length and branching) [22]. We showed that pulsed coherent light irradiation at 670 nm further increases neural outgrowth on this substrate, confirming a favourable effect of laser light irradiation (820 nm) on cell attachment [23]. A similar effect of laser irradiation on neurite outgrowth has been described in microexplants of the brain cortex of adult rats. In these experiments, a He-Ne low power laser irradiation (0.3 mW, 632.8 nm, two 8-min doses, 3.6 J/cm² on two successive days), caused a significant sprouting of cellular processes outgrowth compared to non-irradiated controls in embryonic as well as adult cells [24,25]. Moreover, Higushi at al [26-28] extensively proved that light irradiation influences neurite outgrowth in PC12 cells depending on wavelengths. However, these experiments involve an energy transfer to the cell (0,2; 0,4; 2,5 mW/cm^2^), that is 2000–40000 times higher than energy transfer used in our experimental conditions, so that comparison with our results is not possible. In vivo neurite outgrowth is a contact-dependent process. The regulation that we obtained using an extremely low energy transfer could result from a different synthesis and/or membrane distribution of adhesion molecules, which binds laminin (e.g. integrins). The light is in fact able to regulate both short and long term processes involved in cell contact. Low-power laser light irradiation (632 nm) is able to rapidly remodel cytoskeleton and adhesion structures [29], whereas ultraviolet light regulates integrin expression, thus affecting cell adhesion [30]. Further experiments are needed to approach this point.

### Mitochondria and oxidative stress

The primary events in cells exposed to visible to near-IR radiation are believed to occur in mitochondria [31,7], where one of the three major photoacceptor molecules, e.g. cytochrome c oxidase, is located. Britton Chance's group postulated that about 50% of near infrared light is absorbed by mitochondria chromophores, including cytochome c oxidase [32]. The wavelength used in this study was 670 nm, which corresponds to the absorption spectrum of oxidized cytochrome c oxidase [33]. In this study we used laser-confocal microscopy (excitation blue spectra 488 nm) for the live recording of mitochondria membrane potential (MMP, using JC1 vital dye) under 670 nm light laser irradiation coupled to a classical viability test (MTT assay), proving that short, direct photoirradiation using pulsed red laser light protects against cell death due to oxidative stress through an early mitochondria pathway detected through MMP changes. To our knowledge, this is the first evidence of the neuroprotective effect of red laser irradiation using a live-recording technique. For these experiments, we used the vital dye JC-1. JC-1 monomers rapidly cross the cell membrane and accumulate in the intact mitochondria as aggregates, giving rise to red fluorescence. The brightness of red fluorescence is proportional to ΔΨ [14]. The JC-1 monomer is maximally excited at 490 nm and emits at around 527 nm. When MMP exceeds 140 mV as occurs in dying cells, J-aggregates are formed and the fluorescent emission shifts to 590 nm [34]. Confocal microscopy allows reliable measurement of MMP changes and the time-lapse modality allows the time-course recording of MMP [35]. Using this technique, we recorded MPP changes in single cells from 2 min after stressor exposure (early) to 15–16 min (late), then analyzing these data by variance analysis. MPP variation by H_2_O_2 _exposure is prevented by both short (20 sec) and long (15 min) photoirradiation. Twenty-sec irradiation results in cell viability protection.

A direct beneficial effect of 20 s and 1 min photomodulation using a light emitting diode at 670 nm has been demonstrated in primary neurons exposed to the toxin KCl. This effect has been attributed to the up-regulation of cytochrome c oxidase, which leads to increased energy metabolism and, thus, neuroprotection [36]. Microarray technology also revealed that photobiomodulation by light at 670 nm induced a significant up-regulation of gene expression in pathways involved in mitochondrial energy production and antioxidant cellular protection [37]. This effect is specific to the radiation wavelengths lying between 650- and 680 nm, whereas those lying between 710- and 790 nm reduce photoacceptors [38]. A similar mechanism might be postulated for neuroprotection observed using the 670 nm laser light in PC12 cells exposed to H_2_O_2_. H_2_O_2 _is widely regarded as a cytotoxic agent leading to oxidative stress and mitochondrial dysfunction, whose levels must be minimized by the action of antioxidant defence enzymes [39,40]. Exposure to H_2_O_2 _in the μM range induces a decrease in the mitochondrial transmembrane potential and cytosolic accumulation of the mitochondria cytochrome c, indicating impairment of mitochondrial membrane permeability and reduced cell viability at 4 hr [41].

## Conclusion

We found that laser irradiation affects the in vitro maturation of PC12 cells by stimulating NGF-induced neurite elongation on a laminin-collagen coated substrate. Moreover, coherent light irradiations have a protective effect on oxidative stress induced by H_2_O_2_. Our results demonstrate that 670 nm laser light treatment is neuroprotective and stimulates neural maturation, thus providing additional evidence that red-near-IR light might represent a potential, novel, non-invasive, therapeutic intervention for the treatment of numerous diseases [10].

## Competing interests

Authors **Alessandro Giuliani**, **Alessandro Massella**, **Luciana Giardino**, **Laura Calzà**, declare that they have no competing interest.

Author **Luca Lorenzini**: I do received reimbursements from RGM for the partecipation to two short courses

Author **Michele Gallamini**: BioliteLp020^® ^is a device developed by RGM SpA in close cooperation with La Colletta Bioengineering Center in Genoa Italy. The device is Patented.

Within the framework of a R&D Program co-funded by RGM SpA to verify Biolite effectiveness, in the past years several animal and lab tests have been conducted by the Pathophysiology Center for the Nervous System at Bologna University with the cooperation of the RGM SpA R&D.

The results of the tests have promoted a further expansion of the program to investigate the modifications of the Extracellular Matrix.

RGMD SpA has been established in 2008 to take over and expand the Medical Devices Activities formerly carried out by RGM SpA.

The new research program – codenamed MEDTECH – will be carried out during three years by RGMD SpA in close cooperation with several major Italian Research Centers among which a lead part is played by the BioPharmaNet-Dimorfipa of the High-Technology Laboratory Net at Bologna University.

The program is enjoying a substantial grant from the Italian Ministry of University and Research and is aimed both

• to set up novel diagnostic tools capable to detect and decode physical ECM modifications, and

• to further develop physical means capable to induce ECM modifications as a primary effects to promote therapeutic effects.

An increasing role of the R&D Department of RGMD SpA in all research activities is currently planned.

Authors **Alessandro Giuliani**, **Luca Lorenzini, Michele Gallamini, Massella Alessandro**, **Luciana Giardino**, **Laura Calzà**, declare that they haven't any non-financial competing interests.

## Authors' contributions

AGparticipated in the design of the study, caried out the experimental part on cell culture, MTT and JC-1 assays, and drafted the manuscript. LLpartecipated in the laser exposure and JC-1 assay. MGparticipated in the design of the study and provided the exposure system. AMpartecipated in the MTT assay. LGparticipated in the design of the study and performed the statistical analysis. LCconceived of the study, and participated in its design and coordination and helped to the final version of the manuscript. All authors read and approved the final manuscript.

## Pre-publication history

The pre-publication history for this paper can be accessed here:


